# Abdominal wound closure in the presence of sepsis: our experience with the use of subcutaneous drain

**DOI:** 10.4314/gmj.v58i1.5

**Published:** 2024-03

**Authors:** Esteem Tagar, James Kpolugbo, Andrew E Dongo, Clement Osime, Irekpita Eshiobo, David Irabor

**Affiliations:** 1 Department of Surgery, Irrua Specialist Teaching Hospital, Irrua, Nigeria; 2 Department of Surgery, University of Benin Teaching Hospital, Benin, Nigeria; 3 Department of Surgery, University College Hospital, Ibadan, Nigeria

**Keywords:** Peritonitis, Laparotomy, Surgical Site Infection, Surgical Wound Dehiscence, Suction Drainage

## Abstract

**Objectives:**

Patients requiring surgery for secondary peritonitis demonstrate a significantly increased risk for incisional surgical site infection. This study aimed to evaluate the efficacy of subcutaneous wound drain post-laparotomy for contaminated surgical wounds.

**Design:**

This was a prospective comparative hospital-based study.

**Setting:**

Patients who had surgery for secondary peritonitis in Irrua Specialist Teaching Hospital were studied.

**Participants:**

Fifty patients aged 16 years and above who presented with secondary peritonitis.

**Intervention:**

Patients who met the inclusion criteria were randomized into two equal groups. Group A had a suction drain placed in the subcutaneous space after laparotomy while Group B did not.

**Main outcome measures:**

Development of incisional surgical site infection, wound dehiscence, and duration of post-operative hospital stay.

**Results:**

The incidence of incisional surgical site infection was significantly less in Group A (20%) than in Group B (68%). There was no case of wound dehiscence in Group A as against 3 (12%) in Group B. The difference was not statistically significant. The mean duration of hospital stay was significantly less with subcutaneous suction drain (8.96+2.81 Vs 14.04+8.05; p = 0.005).

**Conclusion:**

Subcutaneous suction drainage is beneficial in abdominal wall closure in cases of peritonitis as it significantly reduces the incidence of incisional surgical site infection and the duration of postoperative hospital stay. The reduction in surgical wound dehiscence observed in this study was, however, not statistically significant.

**Funding:**

None declared

## Introduction

Wounds and their management are fundamental to the practice of surgery. The Center for Disease Control and Prevention (CDC) created a surgical wound classification system to preemptively identify patients at risk of developing surgical site infection (SSI). Among the categories, infection risk ranges from 2% for clean wounds to as high as 30-40% for dirty wounds as seen in cases of abdominal wound closure following surgery for peritonitis. 1 Therefore, patients requiring surgery for peritonitis have a significantly increased risk for surgical site infection. This may lead to wound dehiscence, sometimes progressing to burst abdomen which is often difficult to manage. 2

Surgical site infections are still a major problem in general surgery. They are responsible for significant discomfort for patients and excess morbidity and mortality which often translates into a financial burden on the patient and the health system.^3^ Incisional SSI causes delayed wound healing, bad cosmetic result, prolonged hospital stay, increased cost of treatment and a high risk of developing incisional hernia later on. 4

It has been postulated that the presence of dead space, haematoma, and serous fluid in wounds after laparotomy for peritoneal sepsis increase the risk of surgical site infection as the collection acts as a culture medium. 5 Surgeons have tried many methods to reduce the incidence of wound infection in these patients. Subcutaneous drains have been shown to remove collections and eliminate dead space thus, may result in lowering the rate of wound complications. 6

However, the use of postoperative subcutaneous wound drain is not universally accepted. It is argued that drains may not be efficacious and may cause discomfort and increase hospital stay on their own. 7 Despite the consideration of the usefulness of the subcutaneous drainage method for preventing wound infection, it has been poorly addressed in the literature as there is paucity of randomized controlled trials demonstrating its effectiveness in general abdominal surgery.^8^

This study therefore aims at assessing the efficacy of subcutaneous wound drain in reducing the incidence of incisional SSI and wound complications after laparotomy for secondary peritonitis, in Irrua Specialist Teaching Hospital (ISTH), Irrua, Edo State, Nigeria.

## Methods

### Study design and duration

This was a randomized prospective comparative study of patients with secondary peritonitis who had exploratory laparotomy in Irrua Specialist Teaching Hospital between November 2017 and October 2018.

### Study area

The study was carried out in Irrua Specialist Teaching Hospital, Irrua, Edo State. The hospital is in Edo central senatorial district. It is the biggest tertiary hospital in Edo central senatorial district and receives referrals from various parts of Edo and adjoining States. The study was carried out on the surgical wards (male and female surgical wards), theatre and the microbiology laboratory.

### Eligibility criteria for participants

Patients aged 16 years and above who presented to ISTH, Irrua with secondary peritonitis within the study period formed the study population. They were recruited at presentation to the Accident and Emergency unit or at the time of review on the wards for emergency exploratory laparotomy after obtaining their consent. The exclusion criteria were patients who had prior abdominal surgery before referral to ISTH and re-do laparotomy.

### Sample size determination

The minimum sample size was determined using the formula 9 $n=\frac{2(P)(1-P)(Z_{\beta }+Z\alpha {)}^{2}}{(P_{1}-P_{2}{)}^{2}}$

Where:

n = minimum sample size per group

Z_β_ = the desired power (typically 0.84 for 80% power)

Z_α_ = desired level of statistical significance (typically 1.96)

P_1_ = proportion of patients with wound infection in a study group = 6.45% = 0.06412^10^

P_2_=proportion of patients with wound infection in control group = 51.61% = 0.5161

P = P_1_+P_2_/2 = 0.0645+0.516/2 = 0.2903

n = 2(0.2903) (1−0.2903) (1.96+0.84)^2^/ (0.0645−0.5161)^2^

n = 2(0.2903) (0.7097) (7.84) / (0.2039)

n = 15.84

n = 16 patients per group

10% attrition rate = 16+1.6 = 17.6 = 18

The calculated sample size per group was 18, making a total of 36 patients in both groups. However, 50 patients were recruited for the study.

### Randomization of patients

Based on the calculated sample size, patients who met the inclusion criteria were consecutively enrolled into the study. Simple randomization was used for allocation of patients into two equal groups (A and B). Group ‘A’ had a drain in the subcutaneous space following laparotomy for secondary peritonitis while Group ‘B’ did not.

### Preoperative preparation

The preoperative evaluation included a detailed history, clinical examination, and basic work up for surgery. Optimization of patients to ensure hemodynamic stability was achieved by intravenous fluid resuscitation, correction of electrolyte imbalance and anaemia, nasogastric suction, ensuring adequate urinary output and administration of intravenous antibiotics. Adequate analgesic when indicated. Blood samples were taken for relevant investigations like full blood count, erythrocyte sedimentation rate, serum electrolyte, urea and creatinine, blood grouping and cross matching. Chest radiograph was done to detect radiologic evidence of pneumoperitoneum.Patients had their heights and weights measured preoperatively to calculate the body mass index (BMI).

### Operative technique

Intravenous antibiotics (Ceftriaxone 1g and Metronidazole 500mg) were given at induction of anaesthesia and were continued for 7 days after surgery in their regular dosage in both groups. The choice of this empiric therapy was based on local guideline derived from the surgical infection society revised guidelines on the management of intra-abdominal infections.^11^ Hair at the operation site was shaved preoperatively in the operating room using a surgical blade, after which Chlorhexidine and 70% alcohol solutions were used sequentially in skin preparation of all patients. Exploratory laparotomy was performed through a midline incision. All the surgeons were of consultant and senior registrar grade. The wound edges were wrapped with abdominal packs and the peritoneal collection effectively drained to minimize gross contamination of the wound edges. Peritoneal aspirate was sent for microscopy, culture and sensitivity test. The gastrointestinal pathology was addressed using the procedure best suited for it and the condition of the patient.

The peritoneal cavity was thoroughly irrigated in both groups with warm normal saline and mopped. Closed passive peritoneal drains were used for all patients. Abdominal closure was done using the interrupted mass closure technique with nylon 2, with a suture/wound length ratio of 4:1, and a suture interval of approximately 1cm, taking the fascia at approximately 1.5cm distance from the edge. Wound closure was started from one end of the incision, with all sutures passing through the linea alba and peritoneum. The skin (in groups A and B) was closed with interrupted skin sutures using nylon 2/0. In group A, a subcutaneous closed vacuum drain (Romovac close wound suction unit REF GS-5002) was inserted along the entire length of the incision and brought out through a separate stab wound distally. Effluent volume was recorded daily. The drains were removed when they stopped draining or when the volume of effluent was <5ml in 24 hours. All patients were studied postoperatively for the presence or absence of surgical site infection and wound dehiscence. Temperature, pulse, and blood pressure were checked every 4 hours. Patients' wounds were inspected under aseptic conditions on postoperative days 3, 5 and 7 for local evidence of wound infection and finally at discharge. When discharge was noticed, swab was taken for microscopy, culture, and sensitivity. The diagnostic criteria used for clinical diagnosis of incisional SSI were extrapolations from the Center for Disease Control and Prevention's criteria and include the presence of at least two of the following; erythema (in light complexioned patients), swelling/oedema at the wound margins, discharge of pus/serous effluent from the wound, presence of abnormal odour, presence of tender, inflamed skin and subcutaneous tissue (cellulitis) around the operative wound and the presence of systemic response like fever, tachycardia or tachypnoea in the absence of other possible causes like malaria, blood transfusion reactions. Stitches were removed on postoperative day 10 with evidence of satisfactory wound healing or when wound healing was deemed to be satisfactory to the extent that stitches can be removed. Patients were discharged on assessment of satisfactory recovery from surgery. The results obtained in both groups were compared and analyzed.

### Outcome measures

The primary outcome measure assessed was the proportion of patients who developed incisional SSI in the two groups. The secondary outcome measures included: superficial wound breakdown (defined as skin and/or subcutaneous dehiscence with intact fascial layer), and duration of postoperative hospital stay.

### Data collection

Data collection was done using a predesigned proforma. All relevant information such as biodata, clinical, laboratory and radiological findings were entered into the proforma sheet. Others include the quantity of effluent from the drain every 24 hours, findings on review of incision site on postoperative days 3, 5, 7 and 30, result of microscopy, culture, and sensitivity of discharge from the surgical site, other postoperative complications. Also was assessment of some specific variables that have a possible relationship to postoperative complications such as the operation time, intraoperative blood loss, blood transfusion, BMI, thickness of subcutaneous fat (TSF) and presence or absence of co-morbid medical conditions such as hypertension, diabetes mellitus etc.

### Statistical analysis

Data collected from the study were entered into an electronic spread sheet and analyzed. The statistical analysis was performed using the statistical package for social science (SPSS) (version 21.0, SPSS Inc., Chicago, Illinois). Continuous variables were displayed as the mean ± standard deviation (SD), while the categorical variables were presented as frequencies and percentages. Chi square and Student's t-test were used to test for association when appropriate and a p-value of < 0.05 was considered statistically significant with a confidence level of 95%.

### Ethical considerations

The study was conducted in compliance with the guidelines of the Helsinki declaration on biomedical research in human subjects. Ethical approval was obtained from the Irrua Specialist Teaching Hospital Ethical and Research Committee with reference no: ISTH/REC/20170919/23. The objectives and methods of the study were explained to all the participants. Participation was voluntary, devoid of fear, force, or threat. Confidentiality of the data collected was assured to each respondent by omitting their name and hospital number from the proforma. The risks and benefits of the study were explained to the respondents. Participants were also assured that data obtained from them were strictly meant for research purposes.

## Results

All patients who met the inclusion criteria were recruited and randomized into the two groups (A and B). Group A had subcutaneous drain while Group B did not. The socio-demographic characteristics of all the patients are shown in (Table 1). The age range of participants was 18-90 years, with a mean age of 42.4 ± 15.3 years. The largest proportion of patients (44%) was in the age range of 31-45 years. There were 23 male participants (46%) and 27 female participants (54%) giving a male-female ratio of 1:1.2. Majority of the participants that had subcutaneous drain inserted (76%) were of normal weight while 4%, 12% and 8% of them were underweight, overweight and obese respectively.

**Table 1:** Comparison of sociodemographic and physical characteristics of patients

Variables(s)	Group A Freq (%)	Group B Freq (%)	χ^2^	P-value
**Age (in years)**				
16-30	4(16.0)	5(20.0)	3.054	0.383
31-45	11(44.0)	11(44.0)		
46-60	9(36.0)	5(20.0)		
>60	1(4.0)	4(16.0)		
**Sex**				
Male	10(40.0)	13(52.0)	0.710	0.399
Female	15(60.0)	12(48.0)		
**BMI(Kg/m** ^**2**^ **)**				
Underweight	1(4.0)	3(12.0)	3.691	0.297
Normal	19(76.0)	14(56.0)		
Overweight	3(12.0)	7(28.0)		
Obese	2(8.0)	1(4.0)		
**Subcutaneous fat thickness (cm)**				
<Normal	2(8.0)	2(8.0)	0.511	0.774
Normal	19(76.0)	17(68.0)		
>Normal	4(16.0)	6(24.0)		
**Co-morbidity**				
Yes	6(24.0)	4(16.0)	0.490	0.484
No	19(76.0)	21(84.0)		

*The above table shows that none of the relationships were statistically significant. BMI= Body mass index; χ^2^ = Chi-square test; Freq= Frequency

Most of the participants had normal abdominal subcutaneous fat thickness (72%). However, 20% of them had excessive subcutaneous fat thickness. The mean subcutaneous fat thickness was 2.8 ± 1.0cm.

Forty-six patients (92%) had perforation of viscus with peritonitis while 4 patients (8%) had bowel strangulation with peritonitis (Table 2). The mean operation time (min) ± standard deviation for group A was 109.08 ± 26.04, while group B was 112.12 ± 31.30, with a p-value of 0.711 and the average blood loss was 434 ± 566.37 for group A and 332 ± 243.47 for group B (p = 0.412). Four (16%) of the patients in group A had blood transfusion while only one (4%) of the patients in group B was transfused (p = 0.157). Of the five patients that were transfused, blood transfusion was commenced for two pre-operatively while the remaining three were transfused intraoperatively. None of the patients transfused had surgical site infection.

**Table 2:** Etiology of peritonitis in both groups

Diagnosis at surgery	Group A Frequency (%)	Group B Frequency (%)	χ^2^	P-value
**Perforation**				
**Ruptured Appendix**	11(44.0)	12(48.0)	0.079	0.779
**Duodenal Ulcer**	4(16.0)	3(12.0)	0.163	0.687
**Gastric Ulcer**	7(28.0)	7(28.0)	0.000	1.000
**Trauma induced**	1(4.0)	1(4.0)	0.000	1.000
**Strangulation**				
**Adhesive Small bowel obstruction**	1(4.0)	0(0.0)	1.000	0.317
**Gangrene of terminal ileum from band**	1(4.0)	2(8.0)	0.348	0.556

Nine patients (18%) presented early to the hospital. Majority of the patients (82%) presented late. Thirty-two patients (64%) had laparotomy performed <12 hours of admission while 18(36%) had late intervention. Table 3 reveals a statistically significant difference (p = 0.011) between group A and B with regards to the duration of symptoms before presenting to the hospital. Eight persons (32.0%) in group A presented early as against 1(4.0%) person in group B.

**Table 3:** Duration of symptoms

Variables	SSI Frequency (%)	No SSI Frequency (%)	χ^2^	P-value
**Time of Presentation**				
Early(<24hours)	8(32.0)	1 (4.0)	6.507	0.001*
Late (>24hours)	17(68.0)	24 (96.0)		
**Time of Intervention**				
Early(<12hours)	15(60.0)	17(68.0)	0.340	0.560
Late (>12hours)	10(40.0)	8 (32.0)		

* Statistically significant

**Figure 1:**
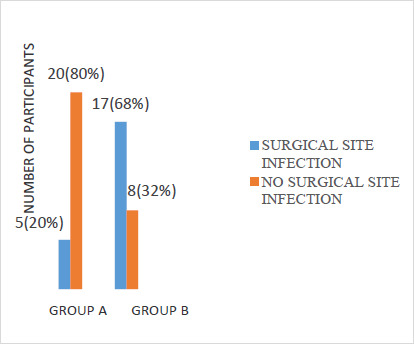
Prevalence of SSI in both groups

**Figure 2:**
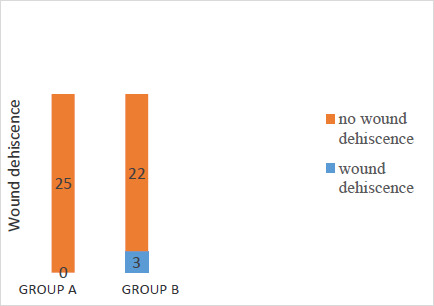
Prevalence of wound dehiscence in both groups

The average drain output from patients in group A was 18.62 ml/day. The drain output was sero-purulent in 20% of patients and serous in 80% of patients. The volume decreased progressively till there was no effluent The median times of removal of suction drain were post-operative day five (range 2-12). The use of subcutaneous closed suction vacuum drain resulted in statistically significant reduction in wound infection (RR=0.29; 95%CI 0.13-0.62, χ^2^=11.455; p=0.001) because, of the 22 participants who had SSI, 5 (20%) were in group A as against 17 (68%) in group B (Fig 1). Twenty patients (90.9%) had superficial SSI while 2 patients (9.1%) had deep. There was no wound breakdown in group A, unlike in group B where 3 (12%) had wound dehiscence, although this was not statistically significant (p = 0.077) [Fig 2]. Age, sex, comorbidity, body mass index, subcutaneous fat thickness, duration of surgery, blood loss during surgery, and blood transfusion were not significant. The regression table below shows the contribution of each of the independent predictive variables in influencing the development of surgical site infection among study participants in both groups. The only predictive variable that influenced the development of surgical site infection was the use of Romovac drain (OR 10.385, 95%CI 2.170- 49.706). Its use was associated with the reduction of SSI in group A. The time of presentation, however, was not significant in the regression model. Therefore, the statistically significant difference in the time of presentation in (Table 3) above did not contribute to the reduced occurrence of SSI among the drain group.

Of the infected wounds, pathogenic organisms were cultured in 20. The most common organism causing SSI in both groups is E Coli. There was no significant difference in the organisms isolated between the two groups. The mean duration of hospital stay for study participants in group A was 8.96 ±2.81 days, while that of group B was 14±8.05 days (p = 0.005) (Table 6).

**Table 6:** Duration of hospital stay in both groups

Variable	Group A	Group B	T	P	95%CI
**Mean hospital stay ± SD (in days)**	8.96+2.81	14.04+8.05	−2.980	0.005*	−8.508 to −1.652

* **Statistically Significant; SD= Standard deviation; T= Student's t-test**

## Discussion

A total of 50 patients with peritonitis were seen during the study period. The preponderance of females in this study was similar to that in a study by Osakwe et al. in Nnewi, in which the females were more (72%) than males (28%), giving a male to female ratio of 1:2.6. 12 This is at variance with the findings in some other studies where there was preponderance of males presenting with this illness. 13,14 The predominance of females in this study may be incidental to this study. This may be due to poor health seeking behavior among males in the locality of this study. 15 The mean age range of the patients was 42.4 ± 15.3 years which is consistent with data available from another study by Patil et al. 16

The absence of statistical correlation between age and outcome, sex and outcome and patients grouping indicate that these variables did not have any confounding influence on the outcome. Obesity, depth of subcutaneous fat, certain comorbidities and operation time have been identified as important factors affecting SSI.^17^,^18^ Allogenic blood transfusion has equally been shown to be associated with an increased risk of SSI when compared to no transfusion or autologous transfusion. The underlying pathophysiological mechanism for this association has not been well-defined but transfusion-associated immunomodulation, in which infusion of circulating antigens present in the transfused blood product lead to a down-regulation of the host immune response has been postulated.^19^ However, in keeping with the conclusions drawn by Berrios-Torres et al. in the CDC guidelines, there is no data to support the withholding of allogenic transfusion in patients as a strategy to prevent SSIs. 20 None of the five patients who had blood transfusion in this study developed SSI. The potential effect of these confounding factors was eliminated from our study, thus, the amelioration of SSI rates in association with subcutaneous suction drain can be reasonably attributed to the beneficial influence of subcutaneous suction drainage.

Empiric antimicrobial therapy is usually initiated as soon as feasible in patients presenting with peritoneal sepsis to reduce the risk of adverse outcomes. This is repeated within one hour before the start of a source control procedure if two half-lives of the agent have passed at the time the intervention is initiated.^11^

Due to the polymicrobial nature of secondary peritonitis, empiric treatment inevitably requires combined treatment to achieve the necessary coverage of both habitual pathogens and unexpected pathogens. We used intravenous ceftriaxone and metronidazole for our patients. The optimal duration of antibiotic therapy is usually individualized and depends on the underlying pathology, severity of infection, speed and effectiveness of source control, and patient response to therapy. In uncomplicated peritonitis in which there is early, adequate source control, a course of 5-7 days of antibiotic therapy is adequate in most cases.^21^

The indications for surgery were similar in both groups (Table 2). Ruptured appendicitis (46%) was the commonest cause of peritonitis in this study, which is in tandem with other studies in the country. 22,23 Ayandipo et al. also reported similar finding of ruptured appendicitis (27.5%) as the commonest cause of peritonitis in their study in Ibadan, Southwest, Nigeria.^13^

The overall infection rate was 44% which is high compared to the rates of SSI reported in some literatures which cite the incidence of SSI following emergency surgeries for peritonitis to be between 20-40%. 16,24 In this study, the incidence of SSI was significantly less in group A (20%) who had subcutaneous drain than in group B (68%) who did not have subcutaneous drain, with a p-value of 0.001. Also, a binary logistic regression analysis revealed that the use of Romovac drain was associated with the reduction of SSI in group A (p = 0.003). On the other hand, the time of presentation was not significant in the regression model (p = 0.531). Among the SSI cases, there was no incidence of wound dehiscence in group A unlike in group B where the incidence of wound dehiscence was 12%, though this was not statistically significant (p = 0.077). The above results are consistent with findings of Sumi et al. in 2014, who retrospectively examined data on 47 patients who underwent emergency operations for colorectal perforation. 25 The clinical features of these cases with or without the use of the J-VAC TM drainage system were examined and statistical analysis was performed. In these high-risk cases, the overall incidence of incisional surgical site infection was 36.2%. The incidence of incisional SSI in these cases with and without the J-VAC _™_ drainage system was 16.7% and 56.5% respectively. These results are similar to that observed in our study. The results suggest that a subcutaneous closed suction drain was effective for preventing incisional SSI in patients who have undergone emergency operations for colorectal perforation. A similar study carried out by Manoharan et al. in 2018 reported 23% infection in the suction drain group and 60% in the group without drain with a p-value of 0.003.

Among the SSI cases, the incidence of wound dehiscence was also significantly less in the drain group (43%) than in the group without drain (89%) with a p-value of 0.015. 26 In January 2019, Wani et al. in India also observed similar results when they carried out a study on 300 patients. They observed SSI in 15.3% of cases with subcutaneous drain and 30% in those without subcutaneous drain with a p-value of 0.002, a result that was also like that of this study. They reported wound dehiscence in 12 % of the cases with subcutaneous drain and 45.3% in the group without subcutaneous drain with a p-value of < 0.001. 27

In our study, we detected SSI as early as on the 3 ^rd^ postoperative day in 80% of the patients with subcutaneous drain as the sero-purulent collection from the drain was sent early for microscopy, culture, and sensitivity whereas, in the patients without drain, 55% of the SSI cases were detected as early as on the 5^th^ postoperative day by the presence of wound discharge. Similar results were seen in the study conducted by Manoharan et al. who found the SSI detection rate as 86% on the 2^nd^ postoperative day and 56% on the 5^th^ postoperative day in patients with and without subcutaneous drain placement respectively. 26 Enteric gram- negative bacteria have previously been reported to be associated with severe SSI. 28 The most common organism causing SSI in this study was found to be Escherichia Coli accounting for 36.4% and 63.6% of cases in drain group and the no-drain group respectively. Most of the SSIs in the group without drain were managed by opening the wound, regular wound dressing and use of antibiotics depending on the culture and sensitivity report. This wound infection resulted in increased morbidity, wound disruption, patient discomfort, poor cosmetic outcome, prolonged hospital stay and increased cost of treatment. On the other hand, cases with drain who developed SSI were easily managed without the need of opening the wound thereby resulting in the reduction of the above challenges.

The mean duration of hospital stay was 8.96±2.81 days for patients who had drain, and 14±8.05 days for those without drain, with a p-value of 0.005. Thus, the postoperative hospital stay was significantly more in group B patients than in group A. This is in tandem with results of previous similar studies where the mean duration of hospital stay was significantly less when subcutaneous suction drain was placed. 2,28 The reason for the shorter hospital stay in the group that had subcutaneous suction drain inserted was as a result of the reduced incidence of surgical site infection and other wound complications. However, some other studies failed to show the beneficial effect of subcutaneous wound drainage. Baier et al.^7^ and Nasta et al.^29^ could not demonstrate a statistically significant reduction in the incidence of surgical site infection post laparotomy by using subcutaneous suction drains in their studies.

The conflicting results from these studies could be attributed to discrepancy in timing of removal of the subcutaneous suction drains postoperatively.

The limitation of this study is that it is from a single centre and the sample size is relatively small for an objective generalization. A large-scale multicenter study is therefore required to support the veracity of these results for reasonable generalization to be possible.

## Conclusion

Peritonitis is a life-threatening surgical emergency with diverse causes. Surgical exploration with peritoneal toileting in addition to source control remains the cornerstone in its management. Postoperatively, surgical site infection is commonly due to endogenous infection from the peritoneal cavity rather than hospital acquired cross infection. Subcutaneous suction drainage is an effective inclusion to abdominal wall closure in cases of peritonitis when compared to conventional primary skin closure without drain, as it significantly reduces the incidence of wound infection (20% Vs 68%; p = 0.001) and the duration of postoperative hospital stay (8.96+2.81 Vs 14.04+8.05; p = 0.005). It also results in a reduction in SSI related complications like wound dehiscence. Although, wound dehiscence was only observed in subjects without closed subcutaneous suction drainage, its occurrence was however not statistically significant. We therefore recommend that subcutaneous drain placement should be considered in abdominal wall closure in patients who undergo emergency surgery for secondary peritonitis.

## Figures and Tables

**Table 4:** Binary logistic regression of predictive factors influencing the occurrence of SSI

Variables	B	P -value	Exp(B)	95%CI of Exp(B)
**Use of Romovac drain**				
Yes	2.340	0.003*	10.385	2.170- 49.706
No	Reference			
**Time of Presentation**				
Early(<24hours)	0.770	0.531	2.161	0.194- 24.036
Late (>24hours)	Reference			

* Statistically significant; Exp= Exponential; CI: Confidence interval

**Table 5:** Distribution of organisms in both groups

Culture yield	Group A (N=5) Frequency (%)	Group B (N=17) Freq (%)	χ^2^	P-value
**E. coli**	4 (80.0)	7 (41.2)	2.224	0.136
**GPC in pairs (+++)**	0 (0.0)	4 (23.5)	1.438	0.535
**Klebsiella spp.**	0 (0.0)	2 (11.8)	0.647	1.000
**Mixed growth**	0 (0.0)	3 (17.6)	1.022	0.558
**No growth**	1 (20.0)	1 (5.9)	0.932	0.411

**E. coli= Escherichia coli; GPC= Gram positive cocci; Spp= Species**				
